# Why do placentas evolve? Evidence for a morphological advantage during pregnancy in live-bearing fish

**DOI:** 10.1371/journal.pone.0195976

**Published:** 2018-04-16

**Authors:** Mike Fleuren, Elsa M. Quicazan-Rubio, Johan L. van Leeuwen, Bart J. A. Pollux

**Affiliations:** Experimental Zoology Chair Group, Department of Animal Sciences, Wageningen University & Research, Wageningen, The Netherlands; Texas A&M University System, UNITED STATES

## Abstract

A live-bearing reproductive strategy can induce large morphological changes in the mother during pregnancy. The evolution of the placenta in swimming animals involves a shift in the timing of maternal provisioning from pre-fertilization (females supply their eggs with sufficient yolk reserves prior to fertilization) to post-fertilization (females provide all nutrients via a placenta during the pregnancy). It has been hypothesised that this shift, associated with the evolution of the placenta, should confer a morphological advantage to the females leading to a more slender body shape during the early stages of pregnancy. We tested this hypothesis by quantifying three-dimensional shape and volume changes during pregnancy and in full-grown virgin controls of two species within the live-bearing fish family Poeciliidae: *Poeciliopsis gracilis* (non-placental) and *Poeciliopsis turneri* (placental). We show that *P*. *turneri* is more slender than *P*. *gracilis* at the beginning of the interbrood interval and in virgins, and that these differences diminish towards the end of pregnancy. This study provides the first evidence for an adaptive morphological advantage of the placenta in live-bearing fish. A similar morphological benefit could drive the evolution of placentas in other live-bearing (swimming) animal lineages.

## Introduction

The placenta, defined as an intimate apposition or fusion of maternal and foetal tissues for physiological exchange [1], has evolved many times independently throughout the animal kingdom (e.g. in invertebrates, fish, amphibians, reptiles and mammals; [2–6]), including at least eight times within the live-bearing fish family Poeciliidae [7–10]. Despite the repeated emergence of placentas among widely diverged animal lineages, it is still unclear what selective forces drive the evolution of placental organs. Three non-mutually exclusive adaptive hypotheses have been proposed to explain why the placenta may have evolved in Poeciliid fish: the resource availability hypothesis, the life history facilitation hypothesis and the locomotor cost hypothesis.

The resource availability hypothesis suggests that the evolution of the placenta and associated reduction in egg size at fertilization might allow females to attain a higher fitness through increased litter sizes. A critical assumption of this hypothesis is that females must be able to abort embryos when facing adverse food conditions [11]. Recent empirical studies in Poeciliidae, however, show that they are not able to do this, suggesting that the conditions under which the placenta might be favoured by natural selection are, at least in this taxonomic group, restricted to environments characterized by high and stable resource conditions [12–15].

The life history facilitation hypothesis states that the placenta might evolve to facilitate the evolution of other life history traits, for example to enable organisms to mature at an earlier age or to produce more or larger offspring that have a higher early-life survivorship [16–21]. However, recent studies in Poeciliidae show that there are no consistent associations between placentation and life history traits, arguing against this hypothesis as a likely explanation for the evolution of the placenta in this taxonomic group [22–24].

**Fig 1:**
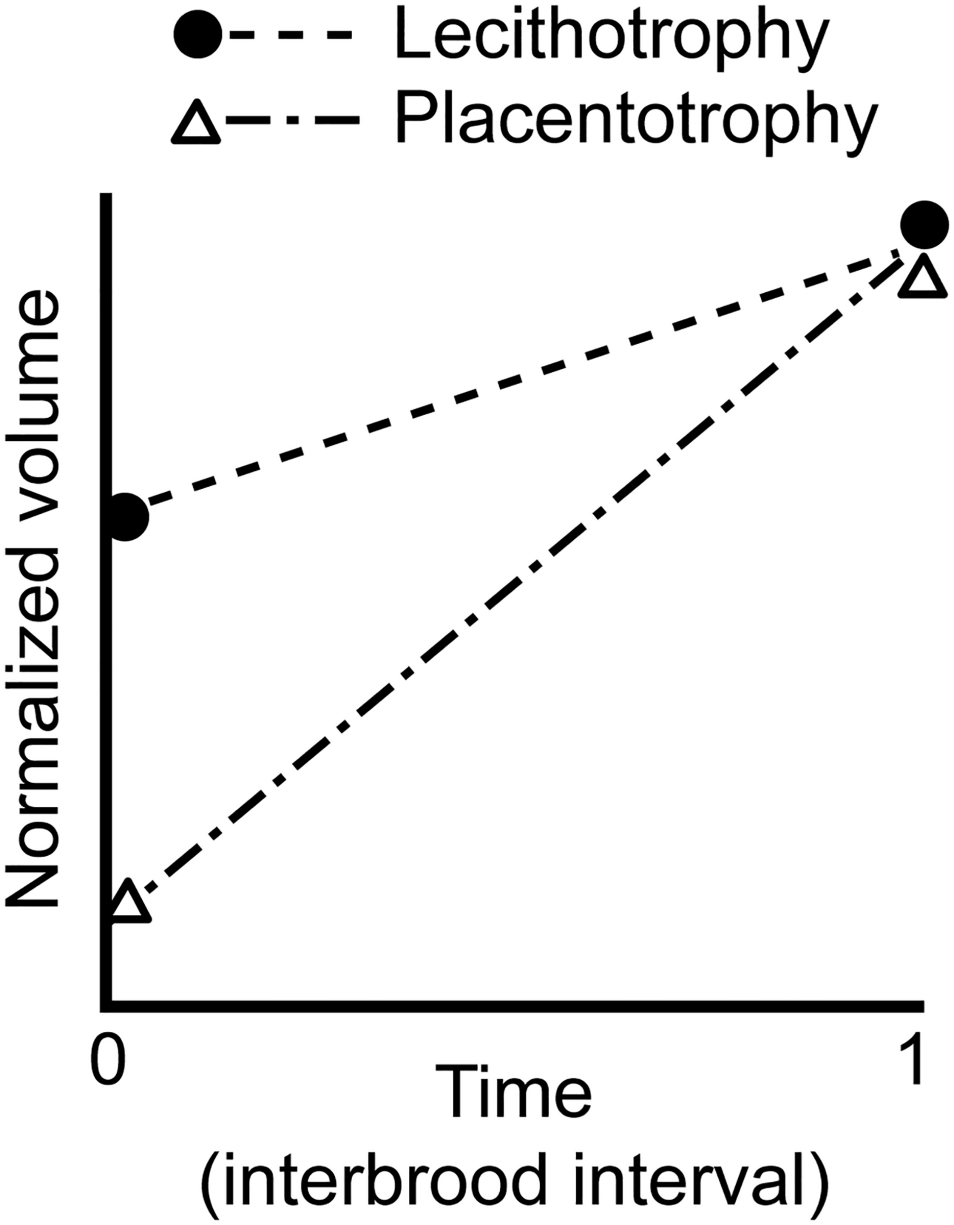
Predicted change in female body volume during pregnancy in two hypothetical lecithotrophic (non-placental) and placentotrophic (placental) live-bearing fish species, assuming an equal female length, offspring number and offspring size at birth (IB = 1): (1) the placental species (dash-dot line) will have a smaller volume during its entire pregnancy than the lecithotrophic species (dashed line) and (2) the relationship for the placental species will show a steeper slope than for the lecithotrophic species, indicating that the difference in body volume will be greatest at the beginning of the pregnancy and gradually diminish towards zero at end of the interbrood interval (redrawn after [9]). Similar plots could be constructed for frontal or wetted surface area. For heuristic purposes the temporal patterns are assumed linear, because the exact shape of the relationship between female volume and time is currently unknown [20].

Finally, the locomotor cost hypothesis argues that the placenta might evolve to offset some of the locomotor cost associated with a live-bearing mode of reproduction. The physical and physiological burden of a pregnancy negatively affects a female’s locomotor performance in a broad range of live-bearing animals (e.g. scorpions, [25]; fishes, [26–29]; reptiles, [30,31]; and mammals, [32,33]). In aquatic animals an increase in abdominal volume may locally limit axial bending and, furthermore, enlarge frontal surface area thereby increasing the drag forces on the body [26,33–35]. An increase in body mass during pregnancy could reduce the ability to rapidly accelerate [27]. It has been postulated that the evolution of the placenta reduces a female’s mean reproductive allotment (RA, the proportion of female mass allocated to developing offspring) during gestation, thereby reducing the distention of the female’s abdomen during the pregnancy without sacrificing her reproductive output [9,36,37]. The argument is that the evolution of the placenta coincides with a shift in the timing of maternal provisioning from pre-fertilization nutrient allocation by building up large amounts of yolk reserves in the eggs prior to fertilization, to the allocation of nutrients after fertilization (via a placental organ throughout the pregnancy). Livebearing species that allocate nutrients prior to fertilization will start with a high RA (and hence a high burden) at the beginning of their pregnancy, because they produce large fully-yolked eggs. Placental species on the other hand species will start with a low RA, because they produce smaller eggs that contain little to no resources and instead rely on nutrient provisioning during gestation. Theory thus predicts that placental females should have a lower reproductive burden (e.g. lower total volume and frontal surface area) at the start of the pregnancy that diminishes over the course of gestation (Fig 1; [9,36,37]). This morphological advantage may improve their locomotor performance (e.g. predator evasion ability; [27]) and hence survival [38] without sacrificing reproductive output, i.e. the locomotor cost hypothesis [9,36,37]. While it is known that Poeciliidae increase in body mass and frontal surface area during pregnancy [26], it is still unclear if, when, and to what extent, the evolution of a placenta alleviates this reproductive burden during pregnancy (Fig 1).

Here, we set out to test the morphological predictions of the locomotor cost hypothesis by comparing body shape changes during gestation in two closely-related sister species within the live-bearing fish genus *Poeciliopsis* (Family Poeciliidae). These species differ markedly in the way they provision their developing embryos: *Poeciliopsis gracilis* lacks a placenta and instead allocates all resources necessary for embryo development to the eggs before fertilization (lecithotrophy), while *Poeciliopsis turneri* has a well-developed placenta (placentotrophy; [7,10]). Specifically, we test whether, compared to *P*. *gracilis*, the placental species *P*. *turneri* has (1) a lower body volume and frontal surface area at the beginning of the interbrood interval and in virgin controls, and (2) a stronger increase in volume and frontal surface area (i.e. have a steeper slope) when pregnancy progresses, indicating that the potentially beneficial reduction in body volume associated with placentation will be greatest at the beginning of the pregnancy and will gradually diminish towards the end of the interbrood interval (Fig 1).

## Material & methods

A detailed description of the used species and their origins, fish rearing protocols and (pre-)experimental husbandry is provided in S1 Text. All procedures were approved by the Animal Ethics Committee of Wageningen University & Research (permit number 2013103). All efforts were made to minimize suffering.

### Time schedule and sample size

We studied changes in body shape during the pregnancy of *Poeciliopsis gracilis* (lecithotrophic) and *Poeciliopsis turneri* (placentotrophic) by creating a series of 3D body reconstructions. For each female, these models were created at evenly spaced time points during one interbrood interval (IB), defined as the period between two parturitions starting the day after a female gave birth (hereafter referred to as IB = 0) and lasting until the next parturition (IB = 1). *Poeciliopsis turneri* was measured every second day and *P*. *gracilis* was measured every fourth day. This served to maintain an approximately equal number of measurements per individual, as the interbrood interval lengths varied between species due to differences in the level of superfetation. Superfetation refers to a reproductive strategy in which females carry multiple broods at different developmental stages [39–41]. Assuming an equal embryo development time, species with a higher level of superfetation (i.e. more simultaneous overlapping broods) will have shorter interbrood intervals (defined as the period between two parturition events) compared to species with lower levels of superfetation [9,42]. Due to the presence of superfetation, IB = 1 does not represent the birth of embryos which eggs were fertilized at IB = 0, but that of an antecedent brood.

To avoid an effect of feeding on body shape (i.e. abdominal extension), the feeding schedules of both species included a 16–24 h food deprivation period prior to the measurements (see S1 Text for further information regarding feeding). Our final dataset comprised 246 three-dimensional body models for 10 pregnant (plus 10 virgin control) *P*. *gracilis* and 14 pregnant (plus 14 virgin control) *P*. *turneri*. Of these 246 data points, six *P*. *turneri* models were omitted preceding analysis because these females were fed shortly before imaging.

### Creation of three-dimensional body models

To create a single body model, a fish was first transferred to a small tank (8 × 8 × 8 cm) with scale bars for image calibration on all walls. Orientation of the female was limited by a separate movable divider. Three photos were taken simultaneously with three Nikon D3200 DSLR cameras (Nikon, Tokyo, Japan; sensor resolution 24 Mpix, equipped with Micro-Nikkor f = 55mm lenses), synchronized with a remote trigger (JinJiaCheng Photography Equipment Co., Ltd., Shenzhen, China) and with LED lights behind glass fibre cloths opposite to the cameras providing diffuse back lighting. The three orthogonally placed cameras yielded a lateral, ventral and rostral/caudal view of the fish. Multiple sets of photos were taken during one measurement session; for further analysis a set of three synchronized pictures was selected in which the fish was in a straight and minimally rotated position.

**Fig 2:**
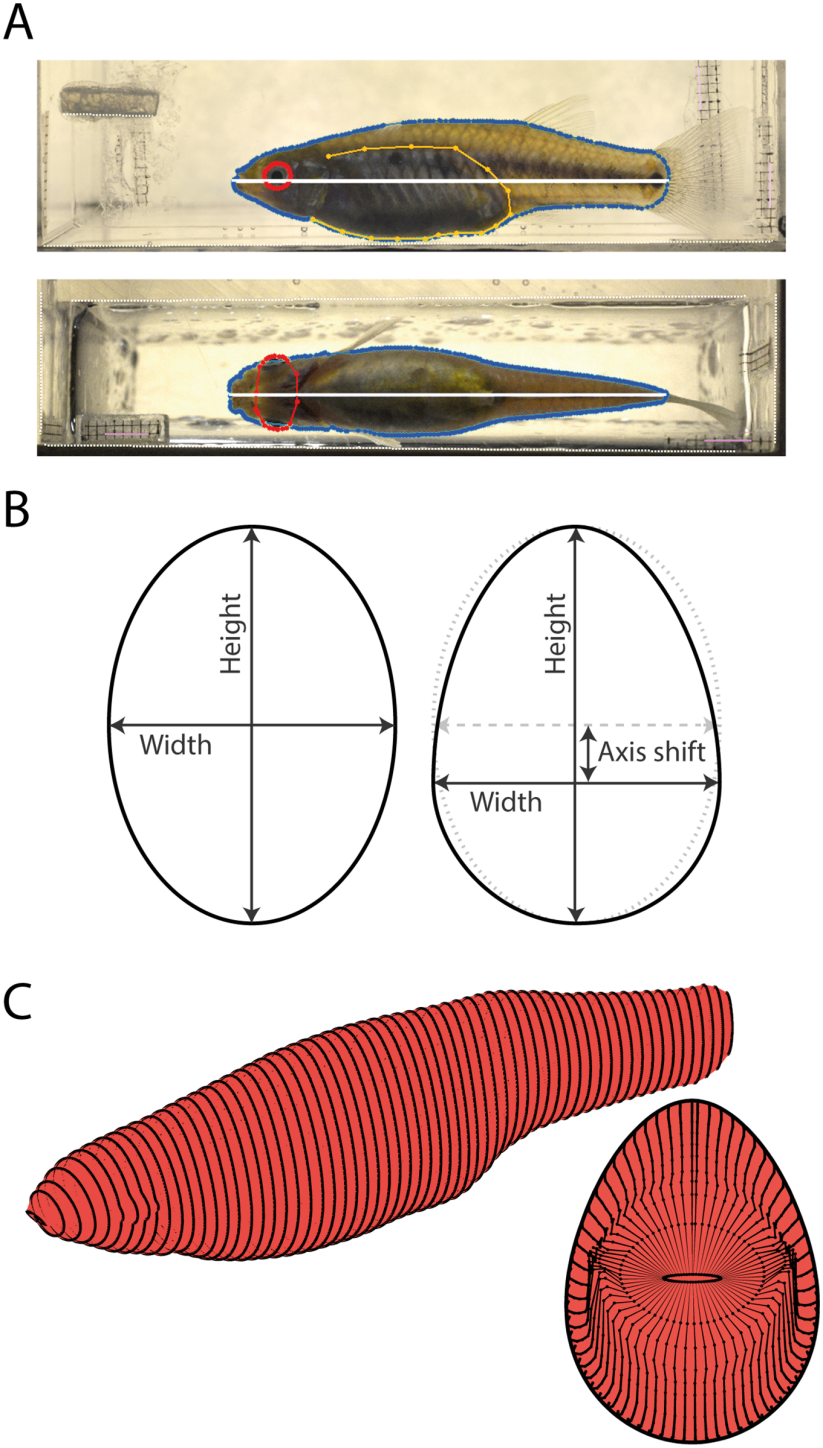
Morphological measurement and 3D model construction. (A) Lateral and ventral photographs in which the trunk (green), abdomen (orange) and eyes (red) are outlined by manually indicated polygons. The longitudinal axis is depicted by white lines. (B) At 251 equidistant points along the longitudinal axis, the width and height of the polygons are converted into ellipse-like cross-sections; in the abdominal area, the vertical position of the horizontal axis is shifted. (C) Stitching the cross-sections of trunk and eyes results in a 3D model from which volume, wetted surface area and frontal surface area (projection at the right) can be calculated. For illustrative purposes these examples only consist of one-fourth of the number of cross-sections.

These photos were subsequently processed with an in-house developed program in MATLAB 2013a (MathWorks, Natick, MA, United States), adapted from a program previously described by [43]. The longitudinal axis of the fish was defined by a straight line between the most anterior point of the snout and the most posterior part of the caudal peduncle (Standard Length, L_SL_; white lines in Fig 2A). The longitudinal axis of the fish consisted of on average 3122 (SE ± 14) pixels and 3193 (± 15) pixels in the lateral and ventral views respectively. Outlines of trunk and eyes were manually digitized (Fig 2A, blue and red lines respectively) as was the position of the abdomen of the fish (lateral view only; delimited by the dorsal edge of the vertebral column, swim bladder and the bottom of the abdomen; Fig 2A, orange line). After cubic spline interpolation of the outlines, the position of the outlines with respect to the longitudinal axis was measured at 251 equidistant points along the longitudinal axis. Using cubic spline interpolation, these points were subsequently converted into ellipse-like cross-sections, that differed in shape depending on whether the section was located in the abdominal region (Fig 2B). In the abdominal region, the minor axis is shifted to half-way the abdominal polygon at that section (default at centre of major axis). Cubic spline interpolations were also used to create a 3D-model of the eyes (with a cubic spline resembling a super-ellipse), which was then stitched to the trunk to create a full 3D-model (Fig 2C).

From these 3D models maximum height, maximum width, frontal surface area (area of frontal view projection, Fig 2C), wetted surface area (total body surface area), and volume were calculated. Maximum height was determined for the whole body, while maximum width was determined for the abdominal region only as the level of opercular distention (the moment in the breathing cycle) caused the position of the widest point to fluctuate in the slender-most fish. These fluctuations only had a minimal effect on the measured total body volume (on average 0.53% with respect to the instance with the least distention). To correct for the effect of intra- and interspecies differences in body size, maximum values of all one-dimensional parameters were normalized by dividing the values by L_SL_, surface areas by dividing by L_SL_^2^ and volume was normalized by dividing by L_SL_^3^.

### Litter wet mass

To get an estimate of the partial reproductive allotment at IB = 1, all new-borns from the litter were caught on the day of delivery and euthanized with a lethal dose of MS-222 (Tricaine-S; Western Chemical Inc., Ferndale, WA, United States). Total litter wet mass was measured after carefully removing excess liquid with a paper towel on a Mettler AE200 analytic balance (scale accuracy 0.0001 g; Mettler-Toledo B.V., Tiel, The Netherlands). Litter wet mass provides a better approximation of reproductive burden than litter dry mass, because the water content of the embryos contributes to the total volume of the brood. Not all *P*. *gracilis* litters could be weighed; however, since offspring size did not differ between females (Mixed model, *F*_9,19_ = 1.31, *P* = 0.2953) total litter wet mass was instead estimated using a linear fit between offspring number and measured litter wet mass (wet mass (g) = 0.0078 ∙ *n*_new-borns_; R^2^ = 0.9469).

### Statistical analysis

The change in morphological parameters was modelled as a two-level longitudinal growth model [44], using the Mixed procedure in SAS version 9.3 (SAS Institute, Cary, NC, United States) under restricted maximum likelihood (REML). This multi-level modelling (MLM) method compares individual growth trajectories between species, allows time to be processed as a continuous variable and is able to handle unbalanced and missing data [44,45]. The model consists of two levels, the level-one model (Eq 1) that represents individual change trajectories, and the level-two model (Eqs 2 and 3) that provides intercept and slope term for the sample average. For each individual (_i_) and time point (_j_), the measured parameter is a function of the individual intercept (α_i_), the individual growth trajectory (*β*_i_ ∙ T_j_) and a random error term for that specific individual and time point (*ε*_*ij*_). Litter wet mass (*w*_*i*_) at IB = 1 was added as a covariate in the level-two model for slope (Eq 3), as arguably larger broods result in increasingly larger morphological parameters due to a higher growth rate. This also allows comparison of the morphologies without the effect of offspring wet mass. The common intercept (*ɤ*_11_), linear slope (*ɤ*_21_) an covariate (*ɤ*_23_) terms in Eqs 2 and 3 represent the values for *P*. *gracilis* while the *ɤ*_12_, *ɤ*_22_ and *ɤ*_24_ terms represent the added difference for *P*. *turneri* for intercept, linear slope and covariate values respectively; *ζ*_*1i*_ and *ζ*_*2i*_ factor individual random error terms. ‘Variance components’ was used as covariance structure (default in SAS Proc Mixed), denominator degrees of freedom were calculated with Kenward-Roger and significance level alpha was set to 0.05 (default in SAS Proc Mixed). To compare model parameter estimates, post-hoc tests were performed using ‘contrast’ and ‘lsmeans’ statements. Virgin data were analysed using a similar MLM method, albeit with a simpler model. Because we did not expect any time-dependent effects, and the virgin controls did not have litter wet mass to use as a covariate, the model consisted solely of an effect of species.

Transformation of the data occasionally resulted in slightly better fits as indicated by marginally higher R^2^-values from linear fits (using Proc GLM in SAS version 9.3), but using transformed data did not change the outcomes of the previously mentioned statistical models. Furthermore, we did not have any *a priori* expectations for the curve of the line. Therefore, we opted to use the original untransformed data and a linear depiction of change.

$$Y_{ij}=\alpha_{i}+{\beta_{i}∙T_{j}+\varepsilon }_{ij}$$ (1)

$$\alpha_{i}=\gamma_{11}+\gamma_{12}∙S_{i}+\zeta_{1i}$$ (2)

$$\beta_{i}=(\gamma_{21}+\gamma_{22}∙S_{i})+{(\gamma }_{23\;}+\gamma_{24}∙S_{i})\;∙w_{i}\;+\zeta_{2i}$$ (3)

## Results

Type 3 tests for Fixed Effects for both the pregnant and the virgin MLM model can be found in S1 Table. All fixed effects in the model were significant, for all measured morphological parameters.

### Morphological changes during pregnancy

**Fig 3:**
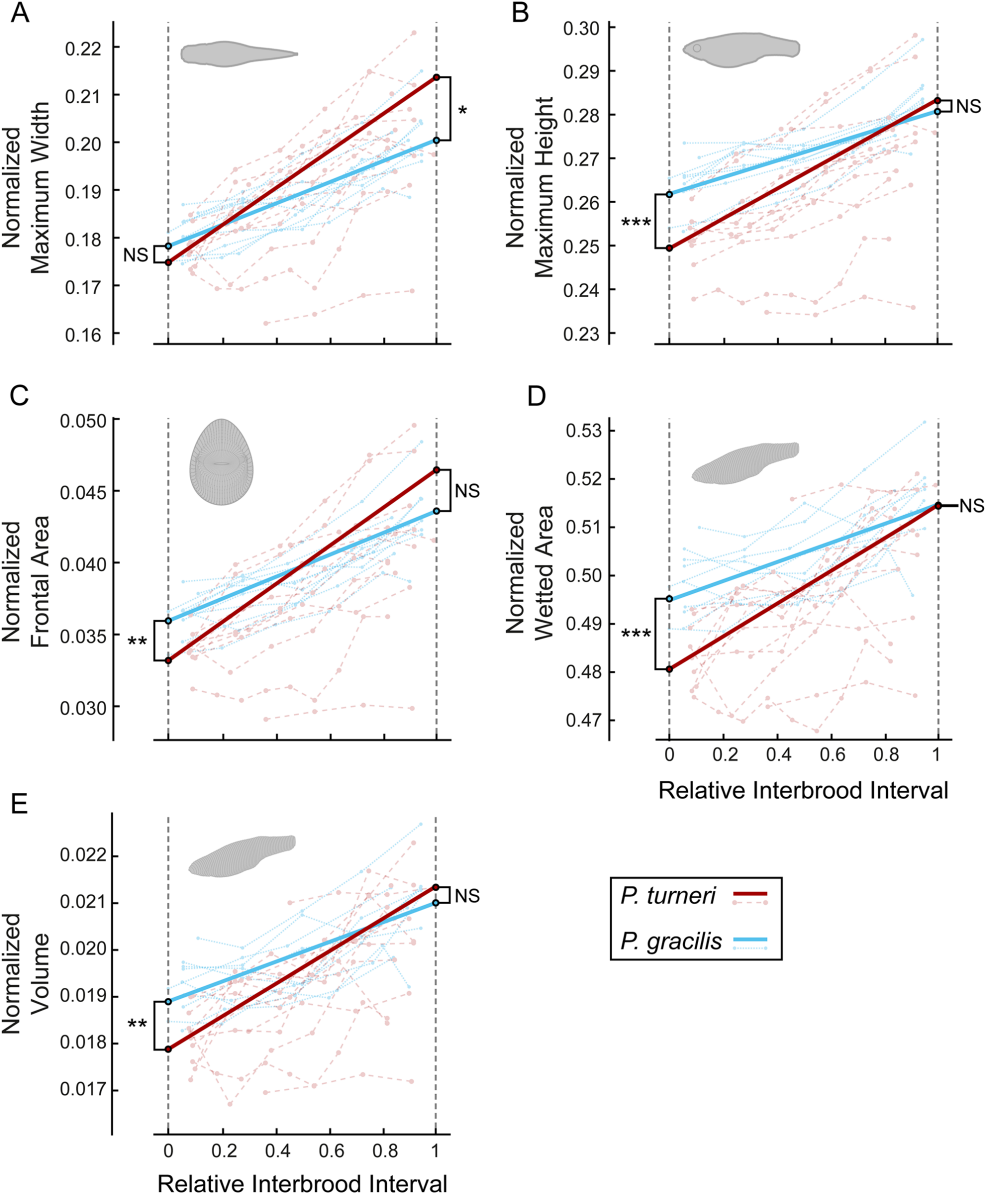
Shape parameters of pregnant *P*. *turneri* (with placenta) and *P*. *gracilis* (without placenta) from *N* = 122 three-dimensional models. The multi-level longitudinal growth models (MLM) indicate changes in normalized (A) maximum width, (B) maximum height, (C) frontal surface area, (D) wetted surface area and (E) volume during one interbrood interval for pregnant *P*. *turneri* (red, *N* = 14) and *P*. *gracilis* (blue, *N* = 10). To account for individual variation in body size, one-dimensional parameters (A and B) were normalized by dividing the values by standard length (L_SL_), the surface areas (C and D) by dividing by L_SL_^2^ and volume (E) by dividing by L_SL_^3^. Connected circles represent individual female growth trajectories, solid lines are plotted from the MLM estimates for intercept and slope with equal litter wet mass (NS = *P* > 0.05, * = 0.01 < *P* < 0.05, ** = 0.001 < *P* < 0.01, *** = *P* < 0.001). Projections show examples of the respective model projections (A–C) or the complete model (D,E).

At the beginning of the interbrood interval (IB = 0), *Poeciliopsis gracilis* females have an overall larger normalized body size than females of *Poeciliopsis turneri*. Except for maximum width (MLM contrast of intercepts: *F*_1,21.2_ = 2.52, *P* = 0.1275; Fig 3A), females of *P*. *gracilis* have a higher maximum height (*F*_1,21.4_ = 15.46, *P* = 0.0007; Fig 3B), frontal surface area (*F*_1,21.6_ = 12.49, *P* = 0.0019; Fig 3C), wetted surface area (*F*_1,24.8_ = 18.17, *P* = 0.0003; Fig 3D) and volume (*F*_1,24.3_ = 12.10, *P* = 0.0019; Fig 3E) than females of *P*. *turneri*.

We found that *P*. *turneri* increases in body size faster than females of *P*. *gracilis*, as indicated by the steeper slopes of the former species in maximum width (MLM contrast of slopes: *F*_1,23_ = 11.42, *P* = 0.0026; Fig 3A), maximum height (*F*_1,17.9_ = 13.01, *P* = 0.0020; Fig 3B), frontal surface area (*F*_1,21.6_ = 11.84, *P* = 0.0024; Fig 3C), wetted surface area (*F*_1,21.1_ = 6.76, *P* = 0.0167; Fig 3D) and volume (*F*_1,22.2_ = 5.92, *P* = 0.0235; Fig 3E). As a consequence, the measured differences at IB = 0 diminished towards the end of pregnancy (IB = 1) (post hoc comparison, maximum height: *P* = 0.6531; frontal surface area: *P* = 0.0866; wetted surface area: *P* = 0.9541; volume: *P* = 0.4696; Fig 3B–3E respectively), while at this point in time females of *P*. *turneri* have a larger maximum width (*P* = 0.0127; Fig 3A). The steeper slopes in *P*. *turneri* are also reflected in the relative increase for maximum width (for *P*. *gracilis* maximum width at IB = 1 is 112% of its value at IB = 0 compared to *P*. *turneri* whose maximum width at IB = 1 is 122% of the value at IB = 0), maximum height (*P*. *gracilis*: 107%, *P*. *turneri*: 113%), frontal surface area (*P*. *gracilis*: 121%, *P*. *turneri*: 140%), wetted surface area (*P*. *gracilis*: 104%, *P*. *turneri*: 107%), and volume (*P*. *gracilis*: 111%, *P*. *turneri*: 120%).

### Morphological differences between virgins

In line with the measured differences at IB = 0 for their pregnant conspecifics, virgin fish of *P*. *gracilis* have a larger overall normalized body size than virgins of *P*. *turneri*. We found significant effects of species on maximum width (MLM contrast: *F*_1,20.9_ = 39.62, *P* < 0.0001; MLM estimate ± SE: *P*.*g*. 0.1800 ± 0.0021, *P*.*t*. 0.1624 ± 0.0018), maximum height (*F*_*1*,*21*.*2*_ = 84.02, *P* < 0.0001; *P*.*g*. 0.2652 ± 0.0025, *P*.*t*. 0.2354 ± 0.0021), frontal surface area (*F*_1,20.6_ = 77.98, *P* < 0.0001; *P*.*g*. 0.0367 ± 0.0006, *P*.*t*. 0.0298 ± 0.0005), wetted surface area (*F*_1,19.5_ = 35.28, *P* < 0.0001; *P*.*g*. 0.4972 ± 0.0032, *P*.*t*. 0.4726 ± 0.0027), and volume (*F*_1,19.3_ = 32.62, *P* < 0.0001; *P*.*g*. 0.0191 ± 0.0003, *P*.*t*. 0.0171 ± 0.0002).

## Discussion

A key aspect of this study is that we compare two different reproductive states (pregnant and virgin fish) in two phylogenetically closely related ‘sister’ species that differ in the way that they provision their embryos [7]: *Poeciliopsis gracilis* is a lecithotrophic species that lacks a placenta, while *Poeciliopsis turneri* represents one of three independent origins of extensive placentation in the genus *Poeciliopsis* [7,10]. *Poeciliopsis gracilis* and *P*. *turneri* have moderate levels of superfetation [23,36]. Due to smaller litters per parturition, higher levels of superfetation could result in reduced litter wet mass (S2 Table). Since the level of superfetation also affects the length of the interbrood interval [9,23,42], time was normalized in our MLM models to account for variation in interbrood interval both within and between species. In absolute terms, the difference in growth rate between the two species would be even more pronounced than is currently shown by our results. Finally, we measured the morphology of virgin controls, because they offer a morphological ‘base-line’ similar to the start of the first pregnancy: all virgin females carried unfertilized, fully yolk-provisioned eggs but were not yet affected by having to carry overlapping broods (superfetation). We showed that virgins of *P*. *turneri* have a more slender body shape than those of *P*. *gracilis*, in line with the observed differences at the beginning of the interbrood interval of pregnant females. At IB = 0, pregnant *P*. *gracilis* are morphologically more alike their virgin conspecifics than pregnant *P*. turneri, probably because the subsequent brood is already further developed in the latter species due to its higher level of superfetation (S3 Table).

Together these findings provide the first evidence in support of two key predictions of the locomotor cost hypothesis that the evolution of post-fertilization maternal provisioning by means of a placenta leads to a more slender body shape at the beginning of the pregnancy, and that this ‘morphological benefit’ diminishes over the course of the pregnancy (Fig 1; [9,36,37]). Our results further show that maximum width is higher in females of *P*. *turneri* during late pregnancy, which could lead to a reduction in abdominal flexibility during this period, more than that experienced by *P*. *gracilis*.

Whether, and to what extent, the measured morphological differences translate directly into differences in swimming performance requires further investigation, as other parameters that determine performance (e.g. physiology, flexural stiffness) could also be affected by pregnancy. Body shape, however, is known to affect the drag forces a fish experiences during swimming, such that more slender animals have a better continuous swimming performance and lower metabolic costs of swimming (e.g. [46–50]). There are two main types of drag that act on swimming fish of this size: pressure drag and friction drag; the former is related to the frontal surface area and the latter to the wetted surface area [51,52]. In *P*. *turneri*, both surface areas are lower at the beginning of pregnancy, and increase more rapidly over time than in *P*. *gracilis* (Fig 3C and 3D), implying an absolute benefit during the early stages and a mean benefit over the whole interbrood interval. During undulatory swimming, the experienced drag is highly complex due to the changing pressure and shear stress distribution on the deforming body [53]. It is, however, apparent that the survival value of optimizing swimming speed and cost of transport (and thus reducing drag forces on the body) could be a driving force behind the evolution of fish morphology [54].

To study female morphology in three dimensions, we used a novel method for collecting longitudinal data, adapted from an in-house developed program originally designed to create 3D-models for zebrafish (*Danio rerio*) larvae [43]. Our method offers two advantages over conventional two-dimensional geometric morphometric approaches. First, we took pictures while the female is in the water, thereby avoiding the need to kill, anesthetize and/or handle the fish with a paintbrush or tweezers [26,55–59]. Minimizing stress, potential physical damage and risk of death is particularly important in longitudinal studies where a single individual is measured repeatedly over a period of time. Second, we use information from 251 equidistant cross-sections and two orthogonal planes to reconstruct 3D body models. Even in a single 2D-plane (e.g. lateral view), our method produces a more accurate approximation of female body shape than landmark-based geometric morphometric approaches, which are often based on a limited number of (semi-)landmarks (typically between 12 to 17) and are hampered by a lack of clear landmarks in the abdominal region of pregnant females [55–59]. Perhaps more importantly, we show that different planes can yield different patterns of shape change through time (e.g. compare the effects in maximum width and height; Fig 3A and 3B), suggesting that information from one plane cannot be readily used to make inferences about temporal changes during the pregnancy in the other planes nor overall streamlining (e.g. volume or frontal surface area).

We studied only one of eight independent origins of the evolution of the placenta in the family Poeciliidae [8,10]. To test the generality of our results, a wider comparative survey is required that includes other independent evolutionary origins of the placenta. Recent studies in the family Poeciliidae have revealed three independent origins of placentation in the genus *Poeciliopsis* (of which this study examined one; [7]) and two independent origins of placentation in the genus *Poecilia* (in the subgenera *Micropoecilia* and *Pamphorichthys*, respectively; [60,61]). These (sub)genera contain closely related species that differ in whether they have a placenta and are eminently suitable for further comparative experimental studies [13,14,23,24,37] to test whether the morphological benefits we found in this study are repeated in other placental lineages.

Moreover, it is possible that drag reduction is one of the driving forces behind the evolution of a placenta in other families of live-bearing bony fish (e.g. Anablepidae, Goodeidae, Zenarchopteridae) [62–64] and of a placenta or other ways of post-fertilization nutrient allocation (matrotrophy) in live-bearing cartilaginous fishes [5,65,66]. Furthermore, it would be worthwhile to study the effects of a placenta on morphology and relevant performance parameters in mobile animals that otherwise try to maximize slenderness, for instance live-bearing Squamate reptiles with a predominantly burrowing or ‘sand-swimming’ mode of locomotion (e.g. viviparous skinks) [67] or animals that are girth-restricted, for instance by the crevices they inhabit [68]. Finally, the placenta evolved many times independently throughout the animal kingdom, in livebearing animal lineages with a large diversity of lifestyles [2–6]; the broader applicability of the locomotor cost hypothesis requires further study.

To conclude, in this study we compared changes in volume and frontal surface area during gestation between a lecithotrophic and a placental fish species using a new 3D-modelling approach. Our results provide the first empirical evidence in support of the locomotor cost hypothesis, which states that the evolution of a placenta can lead to a more slender body shape at the start of the pregnancy and that this effect disappears towards the end of the pregnancy (Figs 1 and 3). To test the generality of our findings, future research should focus on additional independent placental lineages. The biomechanical importance of drag reduction for locomotion, however, suggests that the locomotor cost hypothesis could potentially be applicable to the evolution of placentas in other swimming live-bearing lineages.

## Supporting information

S1 Table Multi-level modelling output for fixed effects in the pregnant and virgin control models, for all measured morphological parameters. LWM: Litter wet mass.
(DOCX)

S2 Table General and reproductive parameters of the experimental fish used in this study. (DOCX)

S3 Table Relative values of morphological parameters of *Poeciliopsis gracilis* and *Poeciliopsis turneri*, compared to their virgin conspecifics. (DOCX)

S1 Text Fish rearing, feeding and husbandry. Detailed description of the study species used, husbandry and feeding.
(DOCX)

S1 Data Spreadsheet containing pregnant fish morphology data. (XLS)

S2 Data Spreadsheet containing virgin fish morphology data. (XLS)
