# Supplementary material for: Why do placentas evolve? Evidence for a morphological advantage during pregnancy in live-bearing fish
Source: PLoS One. 2018 Apr 16;13(4):e0195976. doi: 10.1371/journal.pone.0195976 (PMC5901924; doi:10.1371/journal.pone.0195976)
Supplement: S1 Table — LWM: Litter wet mass. (DOCX) [file pone.0195976.s001.docx]

**Table S1. Multi-level Modelling output for fixed effects in the pregnant and virgin control models, for all measured morphological parameters. LWM: Litter wet mass**

| **Type 3 Test of Fixed Effects** | **Species** | **Species × Time** | **Species × Time × LWM** |
| --- | --- | --- | --- |
|  |  |  |  |
| **Pregnant model** |  |  |  |
| Maximum width | F_2,21.3_ = 11879.6, P < 0.0001 | F_2,23.3_ = 94.60, P < 0.0001 | F_2,19.5_ = 10.11, P = 0.0010 |
| Maximum height | F_2,21.5_ = 12600.0, P < 0.0001 | F_2,18.0_ = 95.08, P < 0.0001 | F_2,17.5_ = 9.95, P = 0.0013 |
| Frontal surface area | F_2,21.7_ = 3978.58, P < 0.0001 | F_2,21.9_ = 99.70, P < 0.0001 | F_2,19.7_ = 12.44, P = 0.0003 |
| Wetted surface area | F_2,25.0_ = 44623.4, P < 0.0001 | F_2,21.4_ = 59.97, P < 0.0001 | F_2,18.3_ = 6.45, P = 0.0076 |
| Volume | F_2,24.5_ = 8327.83, P < 0.0001 | F_2,22.5_ = 60.12, P < 0.0001 | F_2,18.3_ = 7.76, P = 0.0036 |
|  |  |  |  |
| **Virgin model** |  |  |  |
| Maximum width | F_2,20.9_ = 7554.59, P < 0.0001 |  |  |
| Maximum height | F_2,21.3_ = 11952.4, P < 0.0001 |  |  |
| Frontal surface area | F_2,20.6_ = 3620.88, P < 0.0001 |  |  |
| Wetted surface area | F_2,19.5_ = 28014.9, P < 0.0001 |  |  |
| Volume | F_2,19.3_ = 5224.14, P < 0.0001 |  |  |
