# Supplementary material for: Why do placentas evolve? Evidence for a morphological advantage during pregnancy in live-bearing fish
Source: PLoS One. 2018 Apr 16;13(4):e0195976. doi: 10.1371/journal.pone.0195976 (PMC5901924; doi:10.1371/journal.pone.0195976)
Supplement: S2 Table — (DOCX) [file pone.0195976.s002.docx]

**Table S2. General and reproductive parameters of the experimental fish used in this study.**

| **Species** | **Treatment** | ***N*_females_** | **Mean (± SE)**  **SL (m)** | **Mean (± SE)**  **IB interval (days)** | **Mean (± SE)**  ***N*_models per individual_** | **Mean (± SE)**  **Litter wet mass (g)** |
| --- | --- | --- | --- | --- | --- | --- |
| *P. gracilis* | Pregnant | 10 | 0.047 (0.001) ^a^ | 18.3 (0.26) | 4.9 (0.2) | 0.1794 (0.0143) † |
|  | Virgin | 10 | 0.048 (0.001) ^b^ |  |  |  |
|  |  |  |  |  |  |  |
| *P. turneri* | Pregnant | 14 | 0.047 (0.001) ^a^ | 11.9 (0.90) | 5.6 (0.4) | 0.1073 (0.0090) ‡ |
|  | Virgin | 14 | 0.048 (0.001) ^b^ |  |  |  |
|  |  |  |  |  |  |  |

^a,b^ Values with the same superscript not significantly different (MLM, Tukey-Kramer adjusted p-values, alpha < 0.05)

†,‡ Values with the same symbol not significantly different (t-test, t-value = 4.48, df = 22, P = 0.0002)
