# Supplementary material for: Why do placentas evolve? Evidence for a morphological advantage during pregnancy in live-bearing fish
Source: PLoS One. 2018 Apr 16;13(4):e0195976. doi: 10.1371/journal.pone.0195976 (PMC5901924; doi:10.1371/journal.pone.0195976)
Supplement: S3 Table — (DOCX) [file pone.0195976.s003.docx]

**Table S3. Relative values of morphological parameters of *Poeciliopsis gracilis* and *Poeciliopsis turneri*, compared to their virgin conspecifics.** Absolute values can be found in the main text. IB: interbrood interval.

| **Morphological parameter** | ***Poeciliopsis gracilis*** | | |  | ***Poeciliopsis turneri*** | | |
| --- | --- | --- | --- | --- | --- | --- | --- |
|  | **Virgin** | **Pregnant**  (IB = 0) | **Pregnant**  (IB = 1) |  | **Virgin** | **Pregnant**  (IB = 0) | **Pregnant**  (IB = 1) |
|  |  |  |  |  |  |  |  |
| Maximum width | 100 % | 99 % | 111 % |  | 100 % | 108 % | 131 % |
| Maximum height | 100 % | 99 % | 106 % |  | 100 % | 106 % | 120 % |
| Frontal surface area | 100 % | 98 % | 119 % |  | 100 % | 112 % | 157 % |
| Wetted surface area | 100 % | 100 % | 104 % |  | 100 % | 102 % | 109 % |
| Volume | 100 % | 99 % | 110 % |  | 100 % | 105 % | 125 % |
|  |  |  |  |  |  |  |  |
