# Supplementary material for: Why do placentas evolve? Evidence for a morphological advantage during pregnancy in live-bearing fish
Source: PLoS One. 2018 Apr 16;13(4):e0195976. doi: 10.1371/journal.pone.0195976 (PMC5901924; doi:10.1371/journal.pone.0195976)
Supplement: S1 Text — Detailed description of the study species used, husbandry and feeding. (DOCX) [file pone.0195976.s004.docx]

**Supporting Material S1. Fish rearing, feeding and husbandry**

*Study species. – Poeciliopsis gracilis* originated from a small tributary of Rio Motagua, near the village Jones in Zacapa (Guatemala), and *Poeciliopsis turneri* were collected in Rio Purificacion, near Casimiro Castillo, Jalisco (Mexico). These fish stocks were originally housed at the Reznick lab (University of California Riverside, USA). The fish used in the experiments were bred from laboratory stocks that originate from the Reznick lab and are currently held at the Aquatic Research Facilities (ARF) at Wageningen University & Research (the Netherlands).

*Poeciliopsis gracilis* and *P. turneri* are closely related ‘sister’ species that differ markedly in when and how they provision their developing embryos [7]. The matrotrophy index (MI), a dimensionless number defined as the dry mass of the neonate at birth divided by the dry mass of the egg at fertilization, can be used as a proxy for the level of post-fertilization maternal provisioning: *P. gracilis* has an MI of 0.69 (lecithotrophic) and *P. turneri* an MI of 41.4 (placentotrophic, [7]). Both exhibit a moderate degree of superfetation, the presence of multiple broods that differ in the developmental stage of the embryos [24,42], the differences in degree of superfetation are reflected in interbrood interval (mean (± SE) *Pg* 18.3 (± 0.26) days vs. *Pt* 11.9 (± 0.90) days; Table S2).

*Fish rearing & pre-experimental husbandry. –* Breeding stocks were kept in 40 L tanks. From these tanks, new-born juveniles were moved to other stock tanks based on age cohort (age differences within cohorts spanning 2–4 weeks). Sexual maturity was monitored daily and males were removed when sexual characteristics started to develop. Female fish were allowed to grow to a length of approximately 4 cm (± 4–6 months after parturition), after which they were isolated in 9 L isolation tanks (Tecniplast, Bugugiatte, Italy) enriched with gravel and a plastic plant. The water in the isolation tanks was maintained at 24–25ºC and refreshed (~18 l·h^-1^). To ensure homogenisation of water quality among the isolation tanks, all tanks were supplied with water coming from the same biological filtering system. A juvenile conspecific was added to the tanks with virgin *P. gracilis* to reduce potential stress of isolation. The fish were randomly assigned to either a pregnant- or a virgin-treatment group, with one individual of each combined into a ‘measurement block’. One to three males were added to tanks of the pregnant group, and switched around regularly. All companion fish were removed before the start of the experiments. Before the experiments started, the pregnant fish were allowed to go through at least three parturition events, as the first pregnancies are often smaller in brood size and number (Pollux & Reznick, unpublished data). Tanks were checked for new-borns at least once a day and parturition history was meticulously documented. Because of an initial high mortality in the isolated *P. turneri*, 7 additional pregnant females were randomly taken from the stock tanks and included in our study.

*Fish feeding protocol. –* Stock fish were fed recently hatched brine shrimp (*Artemia* nauplii) in the morning (around 8 AM), and either flake paste (TetraMin; Tetra GmbH, Melle, Germany; on Tuesday, Thursday, Saturday and Sunday) or liver paste (Monday, Wednesday and Friday) in the afternoon (around 4 PM). The diet of isolated females consisted of flake- and liver paste during both the mornings and afternoons (days similar to stock), except during the weekend when the females received *Artemia* nauplii. In addition, at the end of each day each isolated female was fed adult brine shrimp. Sixteen to 24 hours prior to the measurements, females were deprived of food to avoid an effect of feeding on body shape (i.e. abdominal extension).
